# 5-[(4-Eth­oxy­anilino)meth­yl]-*N*-(2-fluoro­phen­yl)-6-methyl-2-phenyl­pyrimidin-4-amine

**DOI:** 10.1107/S1600536812021046

**Published:** 2012-05-16

**Authors:** Jerzy Cieplik, Marcin Stolarczyk, Iwona Bryndal, Tadeusz Lis

**Affiliations:** a Department of Organic Chemistry, Medical Academy, 9 Grodzka St, 50-137 Wrocław, Poland; b Faculty of Chemistry, University of Wrocław, 14 Joliot-Curie St, 50-383 Wrocław, Poland; c Department of Bioorganic Chemistry, Faculty of Engineering and Economics, Wrocław University of Economics, 118/120 Komandorska St, 53-345 Wrocław, Poland

## Abstract

The asymmetric unit of the title compound, C_26_H_25_FN_4_O, consists of two symmetry-independent mol­ecules, denoted *A* and *B*. The conformation of each mol­ecule is mainly determined by an intra­molecular N—H⋯N hydrogen bond, which closes a six-membered ring. The dihedral angles between the pyrimidine ring and the phenyl, fluorophenyl and ethoxyphenyl rings are 15.4 (2), 28.4 (2) and 77.5 (2)°, respectively, in mol­ecule *A*, and 15.9 (2), 2.7 (2) and 61.8 (2)° in mol­ecule *B*. Inter­molecular N—H⋯N hydrogen bonds and π–π stacking inter­actions between pyrimidine rings [centroid–centroid distance = 3.692 (4) Å] connect mol­ecules *A* and *B* into dimers and C—H⋯O hydrogen bonds link the dimers into zigzag chains along [011]. The (4-eth­oxy­anilino)methyl group of the *B* mol­ecule is disordered over two sets of sites, the occupancy factor for the major component being 0.900 (2).

## Related literature

For the anti­bacterial activity of 6-methyl-2-phenyl-5-substituted pyrimidine derivatives, see: Cieplik *et al.* (1995 ▶, 2003 ▶, 2008 ▶); Pluta *et al.* (1996 ▶). For related structures, see: Cieplik *et al.* (2006 ▶); Cieplik, Pluta *et al.* (2011 ▶); Cieplik, Stolarczyk *et al.* (2011 ▶).
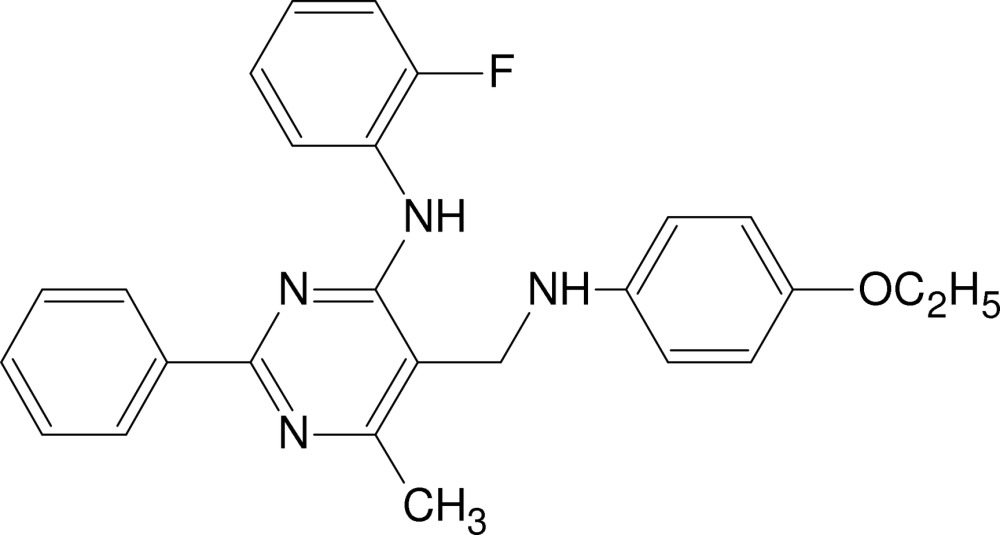


## Experimental

### 

#### Crystal data

C_26_H_25_FN_4_O
*M* *_r_* = 428.50
Triclinic, 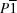

*a* = 9.227 (4) Å
*b* = 10.085 (4) Å
*c* = 23.699 (9) Å
α = 81.92 (4)°
β = 87.52 (4)°
γ = 85.90 (4)°
*V* = 2176.6 (15) Å^3^
*Z* = 4
Mo *K*α radiation
μ = 0.09 mm^−1^
*T* = 85 K
0.55 × 0.05 × 0.05 mm

#### Data collection

Oxford Diffraction Xcalibur PX κ-geometry diffractometer
32959 measured reflections
18015 independent reflections
8365 reflections with *I* > 2σ(*I*)
*R*_int_ = 0.045

#### Refinement

*R*[*F* ^2^ > 2σ(*F* ^2^)] = 0.053
*wR*(*F* ^2^) = 0.079
*S* = 1.00
18015 reflections
620 parameters
H atoms treated by a mixture of independent and constrained refinement
Δρ_max_ = 0.42 e Å^−3^
Δρ_min_ = −0.36 e Å^−3^

### 

Data collection: *CrysAlis CCD* (Oxford Diffraction, 2007 ▶); cell refinement: *CrysAlis RED* (Oxford Diffraction, 2007 ▶); data reduction: *CrysAlis RED*; program(s) used to solve structure: *SHELXS97* (Sheldrick, 2008 ▶); program(s) used to refine structure: *SHELXL97* (Sheldrick, 2008 ▶); molecular graphics: *XP* in *SHELXTL* (Sheldrick, 2008 ▶); software used to prepare material for publication: *SHELXL97*.

## Supplementary Material

Crystal structure: contains datablock(s) I, global. DOI: 10.1107/S1600536812021046/gk2480sup1.cif

Structure factors: contains datablock(s) I. DOI: 10.1107/S1600536812021046/gk2480Isup2.hkl

Supplementary material file. DOI: 10.1107/S1600536812021046/gk2480Isup3.cml

Additional supplementary materials:  crystallographic information; 3D view; checkCIF report

## Figures and Tables

**Table 1:** Hydrogen-bond geometry (Å, °)

*D*—H⋯*A*	*D*—H	H⋯*A*	*D*⋯*A*	*D*—H⋯*A*
N4*A*—H4*A*⋯N5*A*	0.894 (11)	2.093 (11)	2.8465 (17)	141.4 (10)
N5*A*—H5*A*⋯N1*B*^i^	0.879 (11)	2.265 (11)	3.1378 (19)	171.7 (11)
N4*B*—H4*B*⋯N5*B*	0.876 (11)	2.127 (11)	2.8460 (19)	138.9 (10)
N4*B*—H4*B*⋯N5*C*	0.876 (11)	2.184 (15)	2.824 (10)	129.7 (10)
N5*B*—H5*B*⋯N3*A*^i^	0.869 (13)	2.518 (13)	3.350 (2)	160.6 (11)
C46*A*—H46*A*⋯O5*B*	0.95	2.61	3.361 (2)	136
C23*B*—H23*B*⋯O5*A*^ii^	0.95	2.64	3.3437 (19)	132

Symmetry codes: (i) 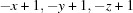; (ii) 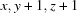.

**Fig. 1.**
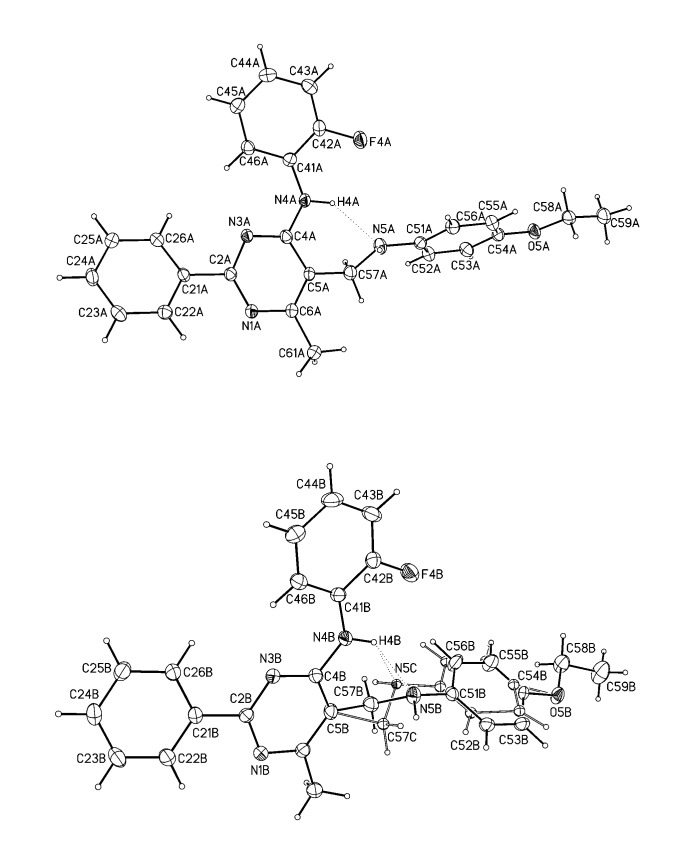
The two symmetry independent molecules of the title compound. Two independent molecules are denoted as A and B (or C in the case of disordered part attached to C5B), respectively. In the part C only two atoms are labelled. Displacement ellipsoids are drawn at the 50% probability level and H atoms are shown as small spheres of arbitrary radii. The dotted lines indicate intramolecular N—H···N hydrogen bonds. Open solid lines represent fragment of a minor component of the disordered B molecule.

**Fig. 2.**
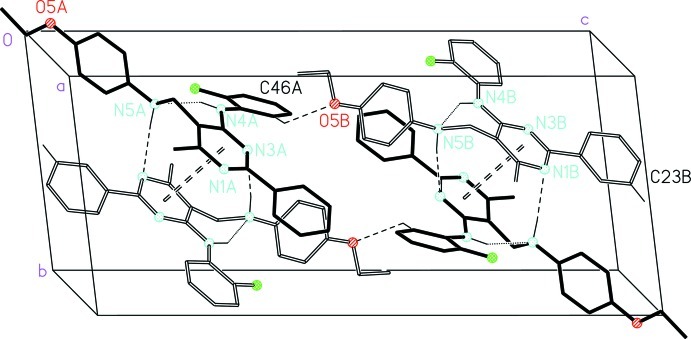
The arrangement of molecules A (solid line) and B (solid open line), showing the intermolecular N—H···N and C—H···O hydrogen bonds (dashed lines) and also the aromatic π-π stacking interactions (double dashed lines). Dotted lines indicate intramolecular hydrogen bonds. The atoms of disordered part C (with the smaller occupancy factor) and H atoms not involved in hydrogen bonding have been omitted for clarity.

## supplementary crystallographic information

### Comment

This work extends the biologial studies and structural data for 6-methyl-2-phenyl-5-substituted pyrimidine derivatives (Cieplik *et al.*, 1995, 2003, 2008; Cieplik, Stolarczyk *et al.*, 2011; Pluta *et al.*, 1996) in connection with the previously characterized two polymorphic forms of *N*-(4-chlorophenyl)-5-[(4-chlorophenyl)aminomethyl]-6-methyl-2-phenylpyrimidin-4-amine (Cieplik *et al.*, 2006) and *N*-(2-fluorophenyl)-5-[(4-methoxyphenyl)aminomethyl]-6-methyl-2-phenylpyrimidin-4-amine (Cieplik, Pluta *et al.*, 2011).

As part of our ongoing studies of this class of derivatives, we have synthesized the title compound, namely *N*-(2-fluorophenyl)-5-[(4-ethoxyphenyl)aminomethyl]-6-methyl-2-phenylpyrimidin-4-amine, and present its structure here.

The asymmetric unit of title crystal consists of two independent molecules, hereafter referred to as A and B (C in case of disordered part attached to C5B), respectively (Fig. 1). The conformation of both molecules is dominated by the dihedral angles between the pyrimidine ring plane and those of the phenyl ring attached to the atom C2 and two other aryl rings of the (2-fluorophenyl)amino or (4-ethoxyphenyl)aminomethyl groups, attached to the atoms C4 or C5 of the pyrimidine ring, respectively (Table 1). These dihedral angles are 15.4 (2), 28.4 (2) and 77.5 (2)° in molecule A and 15.9 (2), 2.7 (2) and 61.8 (2)° in molecule B, respectively.

The molecules of title compound are linked by a combination of N—H···N and C—H···O hydrogen bonds and also the aromatic π–π stacking interactions (Table 2). The C—H···π(arene) hydrogen-bond interactions are absent. The N5 amine atom of molecule A acts as hydrogen-bond donor to the pyrimidine atom N1 of molecule B at (–*x* + 1, –*y* + 1, –*z* + 1). Simultaneously, the amine atom N5 of molecule B acts as hydrogen-bond donor to the pyrimidine atom N3 of molecule A at (-*x* + 1, -*y* + 1, -*z* + 1). This results in the formation of a dimer *via* a cyclic *R*^2^_2_(14) ring motif. Between pyrimidine rings of molecules forming the dimer there is also an aromatic π-π stacking interaction (Fig. 2). The angle between the planes of these rings is 2.84 (8)°. The distance between the ring centroids of the molecules at (*x*, *y*, *z*) and (-*x* + 1, -*y* + 1, -*z* + 1) is 3.692 (4) Å with an interplanar spacing of 3.647 (4) Å and a centroid offset of 0.57 Å. The intermolecular N—H···N interaction was also observed in the polymorphic form of *N*-(4-chlorophenyl)-5-[(4-chlorophenyl)aminomethyl]-6-methyl-2-phenylpyrimidin-4-amine (denoted as I*a*), but the propagation of such linkage generated a chain (Cieplik *et al.*, 2006). In the structure of title compound, intermolecular C—H···O hydrogen bonds between the aryl atoms C46A and C23B and the ethoxy atom O5B and O5A at (*x*, *y* + 1, *z* + 1), respectively (Table 2, Fig. 2) links the molecules into extended zigzag chains which run parallel to the [011] direction and can be described by a C(15) motif.

### Experimental

The title compound was obtained by adopting the procedure described previously by Cieplik *et al.* (2003). 4 g (0.0122 mmol) of 5-(chloromethyl)-*N*-(2-fluorophenyl)-6-methyl-2-phenylpyrimidin-4-amine was dissolved in 50 ml of tetrahydrofuran, and 4.4 g of 4-ethoxyaniline. The reaction mixture was refluxed for 4 h with vigorous stirring, then was cooled and poured into 300 ml of water. The aqueous solution was extracted three times with chloroform (50 ml). The combined chloroform phases were dried over MgSO_4_, filtered and concentrated under vacuum. The oily residue was purified by column chromatography on silica gel (200–300 mesh) using CHCl_3_ as the eluent and by crystallization from methanol to give single crystals (yield: 4.1 g, 78.5%, m.p. 397–398 K).

### Refinement

The N-bonded H atoms were found from difference Fourier maps and refined with *U*_iso_(H) = 1.2 *U*_eq_(N). The remaining H atoms were treated as riding on their carrier atoms, with C—H distances in the range 0.95–0.99 Å, and refined with *U*_iso_(H) = 1.2 *U*_eq_(C), except methyl groups where *U*_iso_(H) = 1.5 *U*_eq_(C). The (4-ethoxyphenyl)aminomethyl group bonded to the pyrimidine C5B atom of the molecule B was found to be disordered over two sites (denoted as B and C). The occupancy factor of the major component (C51B—C57B and N5B) refined at 0.900 (2). The atoms C51C—C57C and N5C were refined with a common isotropic displacement parameter using EADP instruction of *SHELXL97* (Sheldrick, 2008).

### Crystal data

**Table:**

C_26_H_25_FN_4_O	*Z* = 4
*M**_r_* = 428.50	*F*(000) = 904
Triclinic, *P*1	*D*_x_ = 1.308 Mg m^−^^3^
Hall symbol: -P 1	Mo *K*α radiation, λ = 0.71073 Å
*a* = 9.227 (4) Å	Cell parameters from 13387 reflections
*b* = 10.085 (4) Å	θ = 4.2–35.1°
*c* = 23.699 (9) Å	µ = 0.09 mm^−^^1^
α = 81.92 (4)°	*T* = 85 K
β = 87.52 (4)°	Needle, light yellow
γ = 85.90 (4)°	0.55 × 0.05 × 0.05 mm
*V* = 2176.6 (15) Å^3^	

### Data collection

**Table:**

Oxford Diffraction Xcalibur PX κ-geometry diffractometer	8365 reflections with *I* > 2σ(*I*)
Radiation source: normal-focus sealed tube	*R*_int_ = 0.045
Graphite monochromator	θ_max_ = 35.1°, θ_min_ = 4.2°
φ and ω scans	*h* = −14→14
32959 measured reflections	*k* = −16→12
18015 independent reflections	*l* = −36→37

### Refinement

**Table:**

Refinement on *F*^2^	Primary atom site location: structure-invariant direct methods
Least-squares matrix: full	Secondary atom site location: difference Fourier map
*R*[*F*^2^ > 2σ(*F*^2^)] = 0.053	Hydrogen site location: inferred from neighbouring sites
*wR*(*F*^2^) = 0.079	H atoms treated by a mixture of independent and constrained refinement
*S* = 1.00	*w* = 1/[σ^2^(*F*_o_^2^) + (0.016*P*)^2^] where *P* = (*F*_o_^2^ + 2*F*_c_^2^)/3
18015 reflections	(Δ/σ)_max_ = 0.006
620 parameters	Δρ_max_ = 0.42 e Å^−^^3^
0 restraints	Δρ_min_ = −0.36 e Å^−^^3^

### Special details

**Table:**

Geometry. All e.s.d.'s (except the e.s.d. in the dihedral angle between two l.s. planes) are estimated using the full covariance matrix. The cell e.s.d.'s are taken into account individually in the estimation of e.s.d.'s in distances, angles and torsion angles; correlations between e.s.d.'s in cell parameters are only used when they are defined by crystal symmetry. An approximate (isotropic) treatment of cell e.s.d.'s is used for estimating e.s.d.'s involving l.s. planes.
Refinement. Refinement of *F*^2^ against ALL reflections. The weighted *R*-factor *wR* and goodness of fit *S* are based on *F*^2^, conventional *R*-factors *R* are based on *F*, with *F* set to zero for negative *F*^2^. The threshold expression of *F*^2^ > σ(*F*^2^) is used only for calculating *R*-factors(gt) *etc*. and is not relevant to the choice of reflections for refinement. *R*-factors based on *F*^2^ are statistically about twice as large as those based on *F*, and *R*- factors based on ALL data will be even larger.

### Fractional atomic coordinates and isotropic or equivalent isotropic displacement parameters (Å^2^)

**Table:**

	*x*	*y*	*z*	*U*_iso_*/*U*_eq_	Occ. (<1)
N1A	0.92649 (10)	0.40193 (9)	0.25961 (4)	0.0197 (2)	
C2A	0.84388 (12)	0.41558 (10)	0.30609 (4)	0.0174 (2)	
C21A	0.89829 (12)	0.49757 (10)	0.34730 (5)	0.0178 (2)	
C22A	1.01416 (12)	0.57844 (10)	0.33212 (5)	0.0211 (3)	
H22A	1.0577	0.5828	0.2949	0.025*	
C23A	1.06606 (12)	0.65250 (11)	0.37108 (5)	0.0241 (3)	
H23A	1.1443	0.7082	0.3603	0.029*	
C24A	1.00428 (13)	0.64549 (11)	0.42552 (5)	0.0257 (3)	
H24A	1.0400	0.6962	0.4522	0.031*	
C25A	0.89017 (13)	0.56439 (12)	0.44116 (5)	0.0264 (3)	
H25A	0.8481	0.5591	0.4786	0.032*	
C26A	0.83726 (12)	0.49097 (11)	0.40229 (5)	0.0234 (3)	
H26A	0.7588	0.4357	0.4132	0.028*	
N3A	0.71369 (10)	0.36297 (9)	0.31954 (4)	0.0192 (2)	
C4A	0.67114 (12)	0.28197 (10)	0.28397 (5)	0.0191 (2)	
N4A	0.53864 (10)	0.22625 (9)	0.29368 (4)	0.0220 (2)	
H4A	0.5072 (12)	0.2017 (11)	0.2618 (5)	0.026*	
C41A	0.43138 (12)	0.24304 (10)	0.33658 (5)	0.0192 (2)	
C42A	0.28852 (13)	0.22401 (11)	0.32432 (5)	0.0223 (3)	
F4A	0.26553 (7)	0.19057 (7)	0.27156 (3)	0.03230 (18)	
C43A	0.17284 (13)	0.23660 (11)	0.36180 (5)	0.0252 (3)	
H43A	0.0771	0.2242	0.3512	0.030*	
C44A	0.19817 (13)	0.26780 (11)	0.41536 (5)	0.0259 (3)	
H44A	0.1196	0.2788	0.4419	0.031*	
C45A	0.33920 (13)	0.28280 (11)	0.42985 (5)	0.0242 (3)	
H45A	0.3569	0.3018	0.4670	0.029*	
C46A	0.45555 (12)	0.27065 (10)	0.39127 (5)	0.0213 (3)	
H46A	0.5515	0.2812	0.4022	0.026*	
C5A	0.75582 (12)	0.25216 (10)	0.23587 (4)	0.0181 (2)	
C57A	0.70688 (12)	0.15162 (11)	0.20015 (5)	0.0226 (3)	
H5A1	0.7800	0.1397	0.1691	0.027*	
H5A2	0.6984	0.0637	0.2242	0.027*	
N5A	0.56692 (10)	0.19818 (9)	0.17580 (4)	0.0216 (2)	
H5A	0.5650 (12)	0.2849 (11)	0.1640 (5)	0.026*	
C51A	0.50498 (12)	0.12266 (11)	0.13803 (5)	0.0187 (2)	
C52A	0.54689 (12)	−0.01104 (11)	0.13433 (5)	0.0219 (3)	
H52A	0.6213	−0.0560	0.1578	0.026*	
C53A	0.48059 (12)	−0.07958 (11)	0.09651 (5)	0.0230 (3)	
H53A	0.5101	−0.1710	0.0943	0.028*	
C54A	0.37210 (12)	−0.01576 (11)	0.06208 (5)	0.0206 (2)	
C55A	0.32909 (12)	0.11695 (11)	0.06600 (5)	0.0226 (3)	
H55A	0.2546	0.1617	0.0426	0.027*	
C56A	0.39416 (12)	0.18480 (11)	0.10392 (5)	0.0216 (3)	
H56A	0.3625	0.2755	0.1067	0.026*	
O5A	0.31159 (8)	−0.09120 (7)	0.02603 (3)	0.02512 (19)	
C58A	0.20505 (13)	−0.02228 (12)	−0.01139 (5)	0.0277 (3)	
H5A3	0.2484	0.0521	−0.0368	0.033*	
H5A4	0.1231	0.0158	0.0109	0.033*	
C59A	0.15151 (14)	−0.12209 (13)	−0.04635 (5)	0.0340 (3)	
H5A5	0.2342	−0.1634	−0.0664	0.051*	
H5A6	0.0831	−0.0758	−0.0742	0.051*	
H5A7	0.1025	−0.1918	−0.0211	0.051*	
C6A	0.88134 (12)	0.32006 (11)	0.22394 (5)	0.0196 (2)	
C61A	0.97707 (13)	0.30940 (12)	0.17162 (5)	0.0266 (3)	
H6A1	1.0464	0.3796	0.1675	0.032*	
H6A2	0.9170	0.3205	0.1380	0.032*	
H6A3	1.0302	0.2211	0.1753	0.032*	
N1B	0.46939 (10)	0.48944 (9)	0.85823 (4)	0.0196 (2)	
C2B	0.34467 (12)	0.43784 (10)	0.87841 (5)	0.0182 (2)	
C21B	0.29180 (12)	0.46510 (10)	0.93616 (5)	0.0191 (2)	
C22B	0.35471 (13)	0.55961 (12)	0.96319 (5)	0.0269 (3)	
H22B	0.4292	0.6104	0.9438	0.032*	
C23B	0.31006 (13)	0.58056 (12)	1.01793 (5)	0.0303 (3)	
H23B	0.3540	0.6454	1.0358	0.036*	
C24B	0.20219 (13)	0.50776 (11)	1.04662 (5)	0.0272 (3)	
H24B	0.1719	0.5220	1.0842	0.033*	
C25B	0.13823 (13)	0.41375 (11)	1.02032 (5)	0.0275 (3)	
H25B	0.0639	0.3633	1.0400	0.033*	
C26B	0.18195 (12)	0.39280 (11)	0.96545 (5)	0.0233 (3)	
H26B	0.1367	0.3286	0.9476	0.028*	
N3B	0.26406 (10)	0.36164 (8)	0.85164 (4)	0.0184 (2)	
C4B	0.31770 (12)	0.32972 (10)	0.80175 (5)	0.0186 (2)	
N4B	0.23861 (10)	0.25662 (9)	0.77100 (4)	0.0210 (2)	
H4B	0.2722 (12)	0.2512 (11)	0.7362 (5)	0.025*	
C41B	0.10401 (12)	0.20148 (10)	0.78455 (5)	0.0195 (2)	
C42B	0.05870 (13)	0.11807 (11)	0.74759 (5)	0.0225 (3)	
F4B	0.14875 (8)	0.09747 (7)	0.70200 (3)	0.03301 (18)	
C43B	−0.06919 (13)	0.05533 (11)	0.75505 (5)	0.0285 (3)	
H43B	−0.0953	−0.0008	0.7287	0.034*	
C44B	−0.15949 (13)	0.07527 (12)	0.80170 (5)	0.0301 (3)	
H44B	−0.2483	0.0324	0.8079	0.036*	
C45B	−0.11871 (13)	0.15840 (12)	0.83915 (5)	0.0307 (3)	
H45B	−0.1806	0.1726	0.8711	0.037*	
C46B	0.01090 (13)	0.22142 (12)	0.83081 (5)	0.0260 (3)	
H46B	0.0363	0.2787	0.8569	0.031*	
C5B	0.45787 (12)	0.36367 (11)	0.78000 (5)	0.0215 (3)	
C57B	0.52951 (14)	0.29827 (14)	0.73135 (6)	0.0220 (3)	0.900 (2)
H5B1	0.6273	0.3320	0.7226	0.026*	0.900 (2)
H5B2	0.5416	0.2000	0.7429	0.026*	0.900 (2)
N5B	0.44224 (12)	0.32678 (12)	0.67993 (5)	0.0206 (3)	0.900 (2)
H5B	0.4200 (14)	0.4127 (13)	0.6742 (6)	0.025*	0.900 (2)
C51B	0.51109 (15)	0.28230 (15)	0.63039 (6)	0.0174 (3)	0.900 (2)
C52B	0.54393 (15)	0.37379 (14)	0.58272 (6)	0.0198 (3)	0.900 (2)
H52B	0.5183	0.4665	0.5834	0.024*	0.900 (2)
C53B	0.6117 (2)	0.33388 (13)	0.53529 (8)	0.0216 (4)	0.900 (2)
H53B	0.6324	0.3992	0.5035	0.026*	0.900 (2)
C54B	0.6515 (2)	0.19839 (17)	0.53231 (7)	0.0169 (4)	0.900 (2)
C55B	0.61734 (18)	0.10557 (16)	0.57931 (8)	0.0264 (4)	0.900 (2)
H55B	0.6419	0.0128	0.5785	0.032*	0.900 (2)
C56B	0.54733 (18)	0.14772 (15)	0.62757 (6)	0.0271 (4)	0.900 (2)
H56B	0.5240	0.0829	0.6592	0.033*	0.900 (2)
C57C	0.4911 (13)	0.3885 (12)	0.7135 (5)	0.0199 (15)*	0.100 (2)
H5C1	0.4127	0.4455	0.6931	0.024*	0.100 (2)
H5C2	0.5853	0.4290	0.7041	0.024*	0.100 (2)
N5C	0.4955 (12)	0.2544 (11)	0.7018 (5)	0.024 (3)*	0.100 (2)
H5C	0.5534	0.2078	0.7283	0.029*	0.100 (2)
C51C	0.5543 (15)	0.2285 (16)	0.6434 (6)	0.0199 (15)*	0.100 (2)
C52C	0.5467 (17)	0.3303 (16)	0.6028 (7)	0.0199 (15)*	0.100 (2)
H52C	0.5089	0.4174	0.6090	0.024*	0.100 (2)
C53C	0.608 (2)	0.2948 (17)	0.5399 (9)	0.0199 (15)*	0.100 (2)
H53C	0.6124	0.3569	0.5059	0.024*	0.100 (2)
C54C	0.649 (3)	0.170 (2)	0.5445 (9)	0.0199 (15)*	0.100 (2)
C55C	0.6572 (18)	0.0744 (17)	0.5839 (8)	0.0199 (15)*	0.100 (2)
H55C	0.6954	−0.0127	0.5779	0.024*	0.100 (2)
C56C	0.6071 (14)	0.1032 (13)	0.6372 (6)	0.0199 (15)*	0.100 (2)
H56C	0.6101	0.0349	0.6692	0.024*	0.100 (2)
O5B	0.72073 (9)	0.16797 (7)	0.48343 (3)	0.02562 (19)	
C58B	0.74994 (15)	0.02963 (11)	0.47660 (5)	0.0311 (3)	
H5B3	0.6586	−0.0173	0.4810	0.037*	
H5B4	0.8175	−0.0148	0.5058	0.037*	
C59B	0.81682 (17)	0.02406 (13)	0.41799 (6)	0.0454 (4)	
H5B5	0.9088	0.0680	0.4145	0.054*	
H5B6	0.7502	0.0704	0.3894	0.054*	
H5B7	0.8351	−0.0698	0.4118	0.054*	
C6B	0.52680 (12)	0.44986 (10)	0.80905 (5)	0.0198 (2)	
C61B	0.67271 (12)	0.50199 (11)	0.79022 (5)	0.0251 (3)	
H6B1	0.6777	0.5231	0.7486	0.038*	
H6B2	0.7498	0.4335	0.8026	0.038*	
H6B3	0.6855	0.5833	0.8073	0.038*	

### Atomic displacement parameters (Å^2^)

**Table:**

	*U*^11^	*U*^22^	*U*^33^	*U*^12^	*U*^13^	*U*^23^
N1A	0.0194 (5)	0.0241 (5)	0.0164 (5)	−0.0041 (4)	−0.0015 (4)	−0.0032 (4)
C2A	0.0180 (6)	0.0192 (6)	0.0149 (6)	−0.0025 (5)	−0.0023 (5)	−0.0001 (5)
C21A	0.0155 (5)	0.0202 (6)	0.0183 (6)	−0.0019 (5)	−0.0038 (5)	−0.0035 (5)
C22A	0.0203 (6)	0.0226 (6)	0.0205 (6)	−0.0039 (5)	−0.0001 (5)	−0.0019 (5)
C23A	0.0209 (6)	0.0224 (6)	0.0299 (7)	−0.0069 (5)	−0.0024 (5)	−0.0040 (5)
C24A	0.0238 (6)	0.0282 (6)	0.0286 (7)	−0.0026 (5)	−0.0068 (5)	−0.0133 (6)
C25A	0.0234 (6)	0.0364 (7)	0.0217 (7)	−0.0041 (6)	0.0004 (5)	−0.0109 (6)
C26A	0.0187 (6)	0.0305 (6)	0.0225 (6)	−0.0067 (5)	0.0000 (5)	−0.0064 (5)
N3A	0.0194 (5)	0.0231 (5)	0.0162 (5)	−0.0066 (4)	−0.0010 (4)	−0.0035 (4)
C4A	0.0194 (6)	0.0205 (6)	0.0175 (6)	−0.0057 (5)	−0.0026 (5)	0.0002 (5)
N4A	0.0224 (5)	0.0291 (5)	0.0170 (5)	−0.0112 (4)	0.0004 (4)	−0.0073 (4)
C41A	0.0226 (6)	0.0172 (5)	0.0178 (6)	−0.0063 (5)	0.0017 (5)	−0.0007 (5)
C42A	0.0274 (7)	0.0218 (6)	0.0183 (6)	−0.0070 (5)	−0.0043 (5)	−0.0010 (5)
F4A	0.0316 (4)	0.0428 (4)	0.0250 (4)	−0.0160 (3)	−0.0036 (3)	−0.0056 (3)
C43A	0.0207 (6)	0.0243 (6)	0.0299 (7)	−0.0043 (5)	−0.0030 (6)	0.0012 (5)
C44A	0.0243 (7)	0.0234 (6)	0.0291 (7)	−0.0010 (5)	0.0056 (6)	−0.0025 (5)
C45A	0.0294 (7)	0.0220 (6)	0.0215 (6)	−0.0037 (5)	0.0014 (5)	−0.0038 (5)
C46A	0.0219 (6)	0.0199 (6)	0.0230 (6)	−0.0065 (5)	−0.0018 (5)	−0.0027 (5)
C5A	0.0189 (6)	0.0215 (6)	0.0146 (6)	−0.0030 (5)	−0.0028 (5)	−0.0033 (5)
C57A	0.0242 (6)	0.0231 (6)	0.0215 (6)	−0.0033 (5)	−0.0026 (5)	−0.0052 (5)
N5A	0.0245 (5)	0.0178 (5)	0.0237 (6)	−0.0028 (4)	−0.0059 (4)	−0.0050 (4)
C51A	0.0195 (6)	0.0196 (6)	0.0179 (6)	−0.0067 (5)	0.0003 (5)	−0.0039 (5)
C52A	0.0226 (6)	0.0206 (6)	0.0230 (6)	−0.0032 (5)	−0.0058 (5)	−0.0020 (5)
C53A	0.0224 (6)	0.0188 (6)	0.0293 (7)	−0.0026 (5)	−0.0030 (5)	−0.0069 (5)
C54A	0.0208 (6)	0.0237 (6)	0.0195 (6)	−0.0081 (5)	0.0006 (5)	−0.0074 (5)
C55A	0.0214 (6)	0.0239 (6)	0.0229 (6)	−0.0021 (5)	−0.0048 (5)	−0.0031 (5)
C56A	0.0235 (6)	0.0188 (6)	0.0231 (6)	−0.0030 (5)	−0.0017 (5)	−0.0037 (5)
O5A	0.0235 (4)	0.0278 (4)	0.0270 (5)	−0.0044 (4)	−0.0063 (4)	−0.0110 (4)
C58A	0.0207 (6)	0.0384 (7)	0.0268 (7)	−0.0022 (6)	−0.0020 (5)	−0.0139 (6)
C59A	0.0269 (7)	0.0472 (8)	0.0318 (7)	−0.0004 (6)	−0.0054 (6)	−0.0186 (7)
C6A	0.0195 (6)	0.0228 (6)	0.0166 (6)	−0.0018 (5)	−0.0024 (5)	−0.0027 (5)
C61A	0.0242 (6)	0.0356 (7)	0.0220 (6)	−0.0083 (6)	0.0022 (5)	−0.0092 (6)
N1B	0.0219 (5)	0.0191 (5)	0.0182 (5)	−0.0033 (4)	−0.0004 (4)	−0.0035 (4)
C2B	0.0210 (6)	0.0153 (5)	0.0179 (6)	−0.0008 (5)	−0.0023 (5)	0.0001 (5)
C21B	0.0218 (6)	0.0190 (5)	0.0165 (6)	0.0016 (5)	−0.0014 (5)	−0.0032 (5)
C22B	0.0290 (7)	0.0297 (6)	0.0241 (7)	−0.0078 (6)	0.0027 (6)	−0.0092 (6)
C23B	0.0345 (8)	0.0323 (7)	0.0274 (7)	−0.0038 (6)	−0.0030 (6)	−0.0141 (6)
C24B	0.0341 (7)	0.0289 (7)	0.0183 (6)	0.0046 (6)	−0.0010 (6)	−0.0055 (5)
C25B	0.0332 (7)	0.0259 (6)	0.0222 (7)	−0.0024 (6)	0.0053 (6)	−0.0008 (5)
C26B	0.0299 (7)	0.0213 (6)	0.0193 (6)	−0.0034 (5)	0.0003 (5)	−0.0042 (5)
N3B	0.0201 (5)	0.0188 (5)	0.0166 (5)	−0.0027 (4)	0.0006 (4)	−0.0029 (4)
C4B	0.0206 (6)	0.0180 (5)	0.0172 (6)	−0.0025 (5)	−0.0028 (5)	−0.0016 (5)
N4B	0.0210 (5)	0.0271 (5)	0.0165 (5)	−0.0075 (4)	0.0026 (4)	−0.0062 (5)
C41B	0.0194 (6)	0.0192 (6)	0.0201 (6)	−0.0040 (5)	−0.0017 (5)	−0.0015 (5)
C42B	0.0249 (6)	0.0230 (6)	0.0199 (6)	−0.0020 (5)	0.0015 (5)	−0.0045 (5)
F4B	0.0352 (4)	0.0375 (4)	0.0298 (4)	−0.0086 (3)	0.0015 (3)	−0.0141 (3)
C43B	0.0307 (7)	0.0246 (6)	0.0324 (7)	−0.0094 (6)	−0.0080 (6)	−0.0053 (6)
C44B	0.0224 (7)	0.0310 (7)	0.0369 (8)	−0.0116 (6)	−0.0018 (6)	0.0002 (6)
C45B	0.0258 (7)	0.0361 (7)	0.0304 (7)	−0.0093 (6)	0.0059 (6)	−0.0041 (6)
C46B	0.0252 (6)	0.0308 (7)	0.0239 (7)	−0.0088 (5)	0.0012 (5)	−0.0077 (5)
C5B	0.0197 (6)	0.0293 (6)	0.0164 (6)	−0.0045 (5)	0.0020 (5)	−0.0054 (5)
C57B	0.0209 (7)	0.0245 (7)	0.0215 (7)	−0.0043 (6)	−0.0008 (6)	−0.0049 (6)
N5B	0.0231 (6)	0.0215 (6)	0.0168 (6)	0.0024 (5)	−0.0005 (5)	−0.0031 (5)
C51B	0.0162 (7)	0.0214 (7)	0.0153 (7)	−0.0029 (6)	0.0008 (6)	−0.0046 (6)
C52B	0.0231 (7)	0.0163 (7)	0.0204 (8)	−0.0032 (6)	0.0008 (6)	−0.0030 (6)
C53B	0.0277 (8)	0.0157 (8)	0.0212 (8)	−0.0042 (8)	0.0024 (6)	−0.0018 (8)
C54B	0.0214 (7)	0.0196 (10)	0.0100 (9)	−0.0044 (7)	0.0019 (7)	−0.0025 (6)
C55B	0.0385 (11)	0.0166 (9)	0.0223 (8)	0.0032 (7)	0.0058 (8)	−0.0014 (7)
C56B	0.0406 (10)	0.0200 (8)	0.0187 (8)	−0.0028 (7)	0.0084 (7)	0.0019 (6)
O5B	0.0376 (5)	0.0225 (4)	0.0161 (4)	0.0007 (4)	0.0065 (4)	−0.0039 (3)
C58B	0.0446 (8)	0.0230 (6)	0.0248 (7)	0.0028 (6)	0.0065 (6)	−0.0056 (5)
C59B	0.0668 (11)	0.0357 (8)	0.0313 (8)	0.0092 (7)	0.0155 (7)	−0.0077 (6)
C6B	0.0208 (6)	0.0192 (6)	0.0191 (6)	−0.0026 (5)	−0.0014 (5)	−0.0008 (5)
C61B	0.0234 (6)	0.0295 (6)	0.0235 (7)	−0.0077 (5)	0.0008 (5)	−0.0049 (5)

### Geometric parameters (Å, º)

**Table:**

N1A—C2A	1.3298 (15)	C23B—H23B	0.9500
N1A—C6A	1.3585 (14)	C24B—C25B	1.3833 (17)
C2A—N3A	1.3552 (14)	C24B—H24B	0.9500
C2A—C21A	1.4895 (16)	C25B—C26B	1.3851 (16)
C21A—C26A	1.3920 (16)	C25B—H25B	0.9500
C21A—C22A	1.3952 (16)	C26B—H26B	0.9500
C22A—C23A	1.3867 (16)	N3B—C4B	1.3342 (15)
C22A—H22A	0.9500	C4B—N4B	1.3715 (15)
C23A—C24A	1.3824 (17)	C4B—C5B	1.4183 (16)
C23A—H23A	0.9500	N4B—C41B	1.4032 (15)
C24A—C25A	1.3853 (17)	N4B—H4B	0.876 (11)
C24A—H24A	0.9500	C41B—C46B	1.3913 (17)
C25A—C26A	1.3846 (16)	C41B—C42B	1.3925 (16)
C25A—H25A	0.9500	C42B—F4B	1.3643 (14)
C26A—H26A	0.9500	C42B—C43B	1.3717 (17)
N3A—C4A	1.3417 (14)	C43B—C44B	1.3842 (18)
C4A—N4A	1.3788 (15)	C43B—H43B	0.9500
C4A—C5A	1.4092 (16)	C44B—C45B	1.3838 (17)
N4A—C41A	1.4067 (15)	C44B—H44B	0.9500
N4A—H4A	0.894 (11)	C45B—C46B	1.3879 (17)
C41A—C46A	1.3939 (16)	C45B—H45B	0.9500
C41A—C42A	1.3942 (16)	C46B—H46B	0.9500
C42A—F4A	1.3689 (13)	C5B—C6B	1.3854 (16)
C42A—C43A	1.3693 (17)	C5B—C57B	1.5122 (18)
C43A—C44A	1.3833 (16)	C5B—C57C	1.581 (11)
C43A—H43A	0.9500	C57B—N5B	1.473 (2)
C44A—C45A	1.3840 (17)	C57B—H5B1	0.9900
C44A—H44A	0.9500	C57B—H5B2	0.9900
C45A—C46A	1.3896 (17)	C57B—H5C	0.9353
C45A—H45A	0.9500	N5B—C51B	1.4233 (18)
C46A—H46A	0.9500	N5B—H5B	0.869 (13)
C5A—C6A	1.3860 (16)	C51B—C56B	1.3846 (19)
C5A—C57A	1.5127 (16)	C51B—C52B	1.3917 (18)
C57A—N5A	1.4570 (16)	C52B—C53B	1.359 (3)
C57A—H5A1	0.9900	C52B—H52B	0.9500
C57A—H5A2	0.9900	C53B—C54B	1.400 (2)
N5A—C51A	1.4162 (15)	C53B—H53B	0.9500
N5A—H5A	0.879 (11)	C54B—O5B	1.361 (2)
C51A—C56A	1.3891 (17)	C54B—C55B	1.391 (2)
C51A—C52A	1.3896 (16)	C55B—C56B	1.393 (2)
C52A—C53A	1.3919 (16)	C55B—H55B	0.9500
C52A—H52A	0.9500	C56B—H56B	0.9500
C53A—C54A	1.3832 (17)	C57C—N5C	1.416 (16)
C53A—H53A	0.9500	C57C—H5C1	0.9900
C54A—O5A	1.3804 (14)	C57C—H5C2	0.9900
C54A—C55A	1.3831 (16)	N5C—C51C	1.515 (17)
C55A—C56A	1.3836 (16)	N5C—H5C	0.9014
C55A—H55A	0.9500	C51C—C52C	1.304 (18)
C56A—H56A	0.9500	C51C—C56C	1.347 (19)
O5A—C58A	1.4285 (15)	C52C—C53C	1.65 (3)
C58A—C59A	1.5115 (17)	C52C—H52C	0.9500
C58A—H5A3	0.9900	C53C—C54C	1.28 (3)
C58A—H5A4	0.9900	C53C—H53C	0.9500
C59A—H5A5	0.9800	C54C—C55C	1.24 (2)
C59A—H5A6	0.9800	C54C—O5B	1.57 (2)
C59A—H5A7	0.9800	C55C—C56C	1.39 (2)
C6A—C61A	1.5027 (16)	C55C—H55C	0.9500
C61A—H6A1	0.9800	C56C—H56C	0.9500
C61A—H6A2	0.9800	O5B—C58B	1.4322 (14)
C61A—H6A3	0.9800	C58B—C59B	1.5021 (17)
N1B—C2B	1.3386 (15)	C58B—H5B3	0.9900
N1B—C6B	1.3584 (15)	C58B—H5B4	0.9900
C2B—N3B	1.3438 (14)	C59B—H5B5	0.9800
C2B—C21B	1.4885 (16)	C59B—H5B6	0.9800
C21B—C22B	1.3923 (16)	C59B—H5B7	0.9800
C21B—C26B	1.3940 (16)	C6B—C61B	1.5071 (16)
C22B—C23B	1.3842 (17)	C61B—H6B1	0.9800
C22B—H22B	0.9500	C61B—H6B2	0.9800
C23B—C24B	1.3775 (17)	C61B—H6B3	0.9800
			
C2A—N1A—C6A	117.08 (10)	C23B—C24B—H24B	120.2
N1A—C2A—N3A	126.31 (10)	C25B—C24B—H24B	120.2
N1A—C2A—C21A	117.20 (10)	C24B—C25B—C26B	120.37 (12)
N3A—C2A—C21A	116.48 (10)	C24B—C25B—H25B	119.8
C26A—C21A—C22A	118.99 (11)	C26B—C25B—H25B	119.8
C26A—C21A—C2A	120.31 (10)	C25B—C26B—C21B	120.54 (11)
C22A—C21A—C2A	120.66 (11)	C25B—C26B—H26B	119.7
C23A—C22A—C21A	120.34 (11)	C21B—C26B—H26B	119.7
C23A—C22A—H22A	119.8	C4B—N3B—C2B	116.50 (10)
C21A—C22A—H22A	119.8	N3B—C4B—N4B	119.66 (11)
C24A—C23A—C22A	120.15 (11)	N3B—C4B—C5B	122.30 (11)
C24A—C23A—H23A	119.9	N4B—C4B—C5B	117.98 (11)
C22A—C23A—H23A	119.9	C4B—N4B—C41B	129.71 (10)
C23A—C24A—C25A	119.90 (11)	C4B—N4B—H4B	115.0 (7)
C23A—C24A—H24A	120.0	C41B—N4B—H4B	114.9 (8)
C25A—C24A—H24A	120.0	C46B—C41B—C42B	116.56 (11)
C26A—C25A—C24A	120.18 (12)	C46B—C41B—N4B	127.04 (11)
C26A—C25A—H25A	119.9	C42B—C41B—N4B	116.39 (11)
C24A—C25A—H25A	119.9	F4B—C42B—C43B	119.02 (11)
C25A—C26A—C21A	120.43 (11)	F4B—C42B—C41B	117.31 (11)
C25A—C26A—H26A	119.8	C43B—C42B—C41B	123.67 (12)
C21A—C26A—H26A	119.8	C42B—C43B—C44B	118.85 (12)
C4A—N3A—C2A	115.61 (10)	C42B—C43B—H43B	120.6
N3A—C4A—N4A	119.57 (11)	C44B—C43B—H43B	120.6
N3A—C4A—C5A	122.65 (10)	C45B—C44B—C43B	119.15 (12)
N4A—C4A—C5A	117.77 (10)	C45B—C44B—H44B	120.4
C4A—N4A—C41A	129.57 (10)	C43B—C44B—H44B	120.4
C4A—N4A—H4A	111.0 (7)	C44B—C45B—C46B	121.20 (12)
C41A—N4A—H4A	115.6 (7)	C44B—C45B—H45B	119.4
C46A—C41A—C42A	116.81 (11)	C46B—C45B—H45B	119.4
C46A—C41A—N4A	125.98 (11)	C45B—C46B—C41B	120.55 (12)
C42A—C41A—N4A	117.14 (10)	C45B—C46B—H46B	119.7
F4A—C42A—C43A	119.33 (11)	C41B—C46B—H46B	119.7
F4A—C42A—C41A	116.91 (11)	C6B—C5B—C4B	115.81 (11)
C43A—C42A—C41A	123.76 (11)	C6B—C5B—C57B	123.37 (11)
C42A—C43A—C44A	118.66 (11)	C4B—C5B—C57B	120.60 (11)
C42A—C43A—H43A	120.7	C6B—C5B—C57C	112.7 (4)
C44A—C43A—H43A	120.7	C4B—C5B—C57C	120.5 (4)
C43A—C44A—C45A	119.29 (12)	N5B—C57B—C5B	111.22 (11)
C43A—C44A—H44A	120.4	N5B—C57B—H5B1	109.4
C45A—C44A—H44A	120.4	C5B—C57B—H5B1	109.4
C44A—C45A—C46A	121.47 (12)	N5B—C57B—H5B2	109.4
C44A—C45A—H45A	119.3	C5B—C57B—H5B2	109.4
C46A—C45A—H45A	119.3	H5B1—C57B—H5B2	108.0
C45A—C46A—C41A	119.95 (11)	C51B—N5B—C57B	114.39 (11)
C45A—C46A—H46A	120.0	C51B—N5B—H5B	110.9 (9)
C41A—C46A—H46A	120.0	C57B—N5B—H5B	107.7 (9)
C6A—C5A—C4A	116.46 (10)	C56B—C51B—C52B	117.62 (13)
C6A—C5A—C57A	123.66 (11)	C56B—C51B—N5B	121.74 (14)
C4A—C5A—C57A	119.87 (10)	C52B—C51B—N5B	120.64 (12)
N5A—C57A—C5A	110.15 (10)	C53B—C52B—C51B	121.68 (12)
N5A—C57A—H5A1	109.6	C53B—C52B—H52B	119.2
C5A—C57A—H5A1	109.6	C51B—C52B—H52B	119.2
N5A—C57A—H5A2	109.6	C52B—C53B—C54B	121.28 (14)
C5A—C57A—H5A2	109.6	C52B—C53B—H53B	119.4
H5A1—C57A—H5A2	108.1	C54B—C53B—H53B	119.4
C51A—N5A—C57A	119.47 (10)	O5B—C54B—C55B	125.14 (15)
C51A—N5A—H5A	114.4 (7)	O5B—C54B—C53B	117.20 (12)
C57A—N5A—H5A	110.1 (7)	C55B—C54B—C53B	117.67 (17)
C56A—C51A—C52A	118.39 (11)	C54B—C55B—C56B	120.49 (14)
C56A—C51A—N5A	118.15 (10)	C54B—C55B—H55B	119.8
C52A—C51A—N5A	123.45 (11)	C56B—C55B—H55B	119.8
C51A—C52A—C53A	120.43 (11)	C51B—C56B—C55B	121.25 (13)
C51A—C52A—H52A	119.8	C51B—C56B—H56B	119.4
C53A—C52A—H52A	119.8	C55B—C56B—H56B	119.4
C54A—C53A—C52A	120.55 (11)	N5C—C57C—C5B	99.4 (9)
C54A—C53A—H53A	119.7	N5C—C57C—H5C1	111.9
C52A—C53A—H53A	119.7	N5C—C57C—H5C2	111.9
O5A—C54A—C55A	123.95 (11)	H5C1—C57C—H5C2	109.6
O5A—C54A—C53A	116.83 (10)	C57C—N5C—C51C	116.9 (10)
C55A—C54A—C53A	119.22 (11)	C57C—N5C—H5C	104.7
C54A—C55A—C56A	120.25 (11)	C51C—N5C—H5C	108.6
C54A—C55A—H55A	119.9	C52C—C51C—C56C	125.4 (14)
C56A—C55A—H55A	119.9	C52C—C51C—N5C	116.5 (14)
C55A—C56A—C51A	121.14 (11)	C56C—C51C—N5C	118.0 (12)
C55A—C56A—H56A	119.4	C51C—C52C—C53C	114.3 (13)
C51A—C56A—H56A	119.4	C51C—C52C—H52C	122.9
C54A—O5A—C58A	116.27 (9)	C53C—C52C—H52C	122.9
O5A—C58A—C59A	107.95 (10)	C54C—C53C—C52C	108.7 (16)
O5A—C58A—H5A3	110.1	C54C—C53C—H53C	125.6
C59A—C58A—H5A3	110.1	C52C—C53C—H53C	125.6
O5A—C58A—H5A4	110.1	C55C—C54C—C53C	136 (2)
C59A—C58A—H5A4	110.1	C55C—C54C—O5B	124.8 (19)
H5A3—C58A—H5A4	108.4	C53C—C54C—O5B	98.3 (16)
C58A—C59A—H5A5	109.5	C54C—C55C—C56C	115.8 (18)
C58A—C59A—H5A6	109.5	C54C—C55C—H55C	122.1
H5A5—C59A—H5A6	109.5	C56C—C55C—H55C	122.1
C58A—C59A—H5A7	109.5	C51C—C56C—C55C	119.7 (13)
H5A5—C59A—H5A7	109.5	C51C—C56C—H56C	120.1
H5A6—C59A—H5A7	109.5	C55C—C56C—H56C	120.1
N1A—C6A—C5A	121.49 (11)	C54B—O5B—C58B	118.64 (10)
N1A—C6A—C61A	115.12 (10)	O5B—C58B—C59B	107.87 (10)
C5A—C6A—C61A	123.39 (10)	O5B—C58B—H5B3	110.1
C6A—C61A—H6A1	109.5	C59B—C58B—H5B3	110.1
C6A—C61A—H6A2	109.5	O5B—C58B—H5B4	110.1
H6A1—C61A—H6A2	109.5	C59B—C58B—H5B4	110.1
C6A—C61A—H6A3	109.5	H5B3—C58B—H5B4	108.4
H6A1—C61A—H6A3	109.5	C58B—C59B—H5B5	109.5
H6A2—C61A—H6A3	109.5	C58B—C59B—H5B6	109.5
C2B—N1B—C6B	116.76 (10)	H5B5—C59B—H5B6	109.5
N1B—C2B—N3B	125.88 (11)	C58B—C59B—H5B7	109.5
N1B—C2B—C21B	117.13 (10)	H5B5—C59B—H5B7	109.5
N3B—C2B—C21B	116.98 (10)	H5B6—C59B—H5B7	109.5
C22B—C21B—C26B	118.35 (11)	N1B—C6B—C5B	121.92 (11)
C22B—C21B—C2B	120.85 (11)	N1B—C6B—C61B	115.01 (10)
C26B—C21B—C2B	120.76 (10)	C5B—C6B—C61B	123.02 (11)
C23B—C22B—C21B	120.84 (12)	C6B—C61B—H6B1	109.5
C23B—C22B—H22B	119.6	C6B—C61B—H6B2	109.5
C21B—C22B—H22B	119.6	H6B1—C61B—H6B2	109.5
C24B—C23B—C22B	120.30 (12)	C6B—C61B—H6B3	109.5
C24B—C23B—H23B	119.9	H6B1—C61B—H6B3	109.5
C22B—C23B—H23B	119.9	H6B2—C61B—H6B3	109.5
C23B—C24B—C25B	119.60 (12)		
			
C6A—N1A—C2A—N3A	−4.78 (16)	N1B—C2B—N3B—C4B	3.47 (16)
C6A—N1A—C2A—C21A	175.84 (9)	C21B—C2B—N3B—C4B	−175.30 (9)
N1A—C2A—C21A—C26A	−164.85 (10)	C2B—N3B—C4B—N4B	−177.46 (10)
N3A—C2A—C21A—C26A	15.71 (15)	C2B—N3B—C4B—C5B	5.38 (15)
N1A—C2A—C21A—C22A	12.94 (15)	N3B—C4B—N4B—C41B	−2.69 (17)
N3A—C2A—C21A—C22A	−166.50 (10)	C5B—C4B—N4B—C41B	174.59 (10)
C26A—C21A—C22A—C23A	−0.93 (16)	C4B—N4B—C41B—C46B	7.65 (19)
C2A—C21A—C22A—C23A	−178.75 (10)	C4B—N4B—C41B—C42B	−172.53 (11)
C21A—C22A—C23A—C24A	0.73 (17)	C46B—C41B—C42B—F4B	179.99 (9)
C22A—C23A—C24A—C25A	−0.06 (18)	N4B—C41B—C42B—F4B	0.15 (15)
C23A—C24A—C25A—C26A	−0.40 (18)	C46B—C41B—C42B—C43B	−0.82 (17)
C24A—C25A—C26A—C21A	0.19 (18)	N4B—C41B—C42B—C43B	179.34 (10)
C22A—C21A—C26A—C25A	0.48 (17)	F4B—C42B—C43B—C44B	179.27 (10)
C2A—C21A—C26A—C25A	178.30 (11)	C41B—C42B—C43B—C44B	0.10 (18)
N1A—C2A—N3A—C4A	5.01 (16)	C42B—C43B—C44B—C45B	0.43 (18)
C21A—C2A—N3A—C4A	−175.61 (9)	C43B—C44B—C45B—C46B	−0.23 (19)
C2A—N3A—C4A—N4A	−178.62 (10)	C44B—C45B—C46B—C41B	−0.53 (18)
C2A—N3A—C4A—C5A	0.40 (15)	C42B—C41B—C46B—C45B	1.01 (17)
N3A—C4A—N4A—C41A	1.75 (17)	N4B—C41B—C46B—C45B	−179.16 (11)
C5A—C4A—N4A—C41A	−177.32 (10)	N3B—C4B—C5B—C6B	−9.71 (16)
C4A—N4A—C41A—C46A	−29.55 (18)	N4B—C4B—C5B—C6B	173.09 (10)
C4A—N4A—C41A—C42A	153.75 (11)	N3B—C4B—C5B—C57B	165.10 (11)
C46A—C41A—C42A—F4A	−176.97 (9)	N4B—C4B—C5B—C57B	−12.10 (16)
N4A—C41A—C42A—F4A	0.04 (14)	N3B—C4B—C5B—C57C	−151.1 (5)
C46A—C41A—C42A—C43A	2.85 (16)	N4B—C4B—C5B—C57C	31.7 (5)
N4A—C41A—C42A—C43A	179.87 (10)	C6B—C5B—C57B—N5B	−124.97 (13)
F4A—C42A—C43A—C44A	178.72 (9)	C4B—C5B—C57B—N5B	60.62 (15)
C41A—C42A—C43A—C44A	−1.10 (17)	C57C—C5B—C57B—N5B	−40.7 (7)
C42A—C43A—C44A—C45A	−1.22 (16)	C5B—C57B—N5B—C51B	172.76 (10)
C43A—C44A—C45A—C46A	1.70 (16)	C57B—N5B—C51B—C56B	62.82 (17)
C44A—C45A—C46A—C41A	0.13 (16)	C57B—N5B—C51B—C52B	−117.41 (14)
C42A—C41A—C46A—C45A	−2.30 (15)	C56B—C51B—C52B—C53B	−1.3 (2)
N4A—C41A—C46A—C45A	−179.01 (10)	N5B—C51B—C52B—C53B	178.95 (15)
N3A—C4A—C5A—C6A	−5.34 (16)	C51B—C52B—C53B—C54B	0.0 (3)
N4A—C4A—C5A—C6A	173.70 (10)	C52B—C53B—C54B—O5B	−179.11 (17)
N3A—C4A—C5A—C57A	175.24 (10)	C52B—C53B—C54B—C55B	1.0 (3)
N4A—C4A—C5A—C57A	−5.72 (15)	O5B—C54B—C55B—C56B	179.40 (17)
C6A—C5A—C57A—N5A	−117.85 (12)	C53B—C54B—C55B—C56B	−0.7 (3)
C4A—C5A—C57A—N5A	61.52 (14)	C52B—C51B—C56B—C55B	1.5 (2)
C5A—C57A—N5A—C51A	176.32 (9)	N5B—C51B—C56B—C55B	−178.68 (14)
C57A—N5A—C51A—C56A	−163.04 (10)	C54B—C55B—C56B—C51B	−0.6 (3)
C57A—N5A—C51A—C52A	18.54 (16)	C6B—C5B—C57C—N5C	144.2 (6)
C56A—C51A—C52A—C53A	1.11 (16)	C4B—C5B—C57C—N5C	−73.4 (9)
N5A—C51A—C52A—C53A	179.53 (11)	C57B—C5B—C57C—N5C	28.4 (6)
C51A—C52A—C53A—C54A	−0.01 (17)	C5B—C57C—N5C—C51C	−168.3 (9)
C52A—C53A—C54A—O5A	−179.66 (10)	C57C—N5C—C51C—C52C	−24.0 (18)
C52A—C53A—C54A—C55A	−0.59 (17)	C57C—N5C—C51C—C56C	157.3 (12)
O5A—C54A—C55A—C56A	179.07 (10)	C56C—C51C—C52C—C53C	0 (2)
C53A—C54A—C55A—C56A	0.07 (17)	N5C—C51C—C52C—C53C	−178.4 (13)
C54A—C55A—C56A—C51A	1.07 (17)	C51C—C52C—C53C—C54C	1 (2)
C52A—C51A—C56A—C55A	−1.64 (17)	C52C—C53C—C54C—C55C	−3 (4)
N5A—C51A—C56A—C55A	179.86 (11)	C52C—C53C—C54C—O5B	−172.3 (13)
C55A—C54A—O5A—C58A	4.12 (15)	C53C—C54C—C55C—C56C	2 (4)
C53A—C54A—O5A—C58A	−176.86 (10)	O5B—C54C—C55C—C56C	169.6 (16)
C54A—O5A—C58A—C59A	−179.27 (9)	C52C—C51C—C56C—C55C	−1 (2)
C2A—N1A—C6A—C5A	−0.92 (16)	N5C—C51C—C56C—C55C	177.7 (13)
C2A—N1A—C6A—C61A	179.17 (9)	C54C—C55C—C56C—C51C	0 (2)
C4A—C5A—C6A—N1A	5.58 (16)	C55B—C54B—O5B—C58B	6.1 (2)
C57A—C5A—C6A—N1A	−175.03 (10)	C53B—C54B—O5B—C58B	−173.78 (15)
C4A—C5A—C6A—C61A	−174.52 (10)	C55B—C54B—O5B—C54C	−7 (4)
C57A—C5A—C6A—C61A	4.87 (17)	C53B—C54B—O5B—C54C	173 (4)
C6B—N1B—C2B—N3B	−7.16 (16)	C55C—C54C—O5B—C54B	−173 (6)
C6B—N1B—C2B—C21B	171.61 (9)	C53C—C54C—O5B—C54B	−2 (3)
N1B—C2B—C21B—C22B	11.38 (16)	C55C—C54C—O5B—C58B	19 (2)
N3B—C2B—C21B—C22B	−169.73 (11)	C53C—C54C—O5B—C58B	−169.5 (14)
N1B—C2B—C21B—C26B	−166.05 (10)	C54B—O5B—C58B—C59B	176.13 (13)
N3B—C2B—C21B—C26B	12.84 (15)	C54C—O5B—C58B—C59B	179.1 (9)
C26B—C21B—C22B—C23B	0.48 (18)	C2B—N1B—C6B—C5B	2.00 (16)
C2B—C21B—C22B—C23B	−177.02 (11)	C2B—N1B—C6B—C61B	−175.57 (9)
C21B—C22B—C23B—C24B	0.03 (19)	C4B—C5B—C6B—N1B	5.77 (16)
C22B—C23B—C24B—C25B	−0.23 (18)	C57B—C5B—C6B—N1B	−168.88 (10)
C23B—C24B—C25B—C26B	−0.09 (18)	C57C—C5B—C6B—N1B	150.1 (5)
C24B—C25B—C26B—C21B	0.61 (18)	C4B—C5B—C6B—C61B	−176.86 (10)
C22B—C21B—C26B—C25B	−0.79 (17)	C57B—C5B—C6B—C61B	8.49 (18)
C2B—C21B—C26B—C25B	176.71 (10)	C57C—C5B—C6B—C61B	−32.5 (5)

### Hydrogen-bond geometry (Å, º)

**Table:**

*D*—H···*A*	*D*—H	H···*A*	*D*···*A*	*D*—H···*A*
N4*A*—H4*A*···N5*A*	0.894 (11)	2.093 (11)	2.8465 (17)	141.4 (10)
N5*A*—H5*A*···N1*B*^i^	0.879 (11)	2.265 (11)	3.1378 (19)	171.7 (11)
N4*B*—H4*B*···N5*B*	0.876 (11)	2.127 (11)	2.8460 (19)	138.9 (10)
N4*B*—H4*B*···N5*C*	0.876 (11)	2.184 (15)	2.824 (10)	129.7 (10)
N5*B*—H5*B*···N3*A*^i^	0.869 (13)	2.518 (13)	3.350 (2)	160.6 (11)
C46*A*—H46*A*···O5*B*	0.95	2.61	3.361 (2)	136
C23*B*—H23*B*···O5*A*^ii^	0.95	2.64	3.3437 (19)	132
C46*A*—H46*A*···N3*A*	0.95	2.49	2.992 (2)	113
C46*B*—H46*B*···N3*B*	0.95	2.31	2.9100 (19)	121

Symmetry codes: (i) −*x*+1, −*y*+1, −*z*+1; (ii) *x*, *y*+1, *z*+1.
